# Carga sobre os metatarsos menores após cirurgia minimamente invasiva para correção de hallux valgus: Um modelo de elementos finitos

**DOI:** 10.1055/s-0045-1813000

**Published:** 2025-12-10

**Authors:** Henrique Mansur, Bruno Abdo, Gabriel Ferraz Ferreira, Miguel Viana Pereira Filho, Roberto Zambelli, Gustavo Araújo Nunes

**Affiliations:** 1 Departamento de Cirurgia do Pé e Tornozelo, Instituto Return to Play and Instituto D'Or de Pesquisa e Ensino (IDOR), Brasília, DF, Brasil; 2 Departamento de Cirurgia do Pé e Tornozelo, Instituto D'Or de Pesquisa e Ensino (IDOR), Brasília, DF, Brasil; 3 Grupo de Cirurgia de Pé e Tornozelo, Unidade de Ortopedia e Traumatologia, Prevent Senior, São Paulo, SP, Brasil; 4 Chefe do Grupo de Cirurgia de Pé e Tornozelo, Unidade de Ortopedia e Traumatologia, Prevent Senior, São Paulo, SP, Brasil; 5 Faculdade de Ciências Médicas de Minas Gerais, Belo Horizonte, MG, Brasil; 6 Rede de Saúde Mater Dei, Belo Horizonte, MG, Brasil; 7 Unidade de Pé e Tornozelo, Clínica Ortopédica ÓRION, Brasília, DF, Brasil

**Keywords:** análise de elementos finitos, deformidades congênitas do pé, hallux valgus, procedimentos cirúrgicos minimamente invasivos, finite element analysis, foot deformities, congenital, hallux valgus, minimally invasive surgical procedures

## Abstract

**Objetivo:**

Analisar as consequências biomecânicas nos metatarsos menores do uso de diferentes configurações de parafusos para fixação da osteotomia minimamente invasiva em Chevron e Akin (MICA, do inglês *minimally-invasive Chevron-Akin* ) por meio do método dos elementos finitos (MEF).

**Métodos:**

Um modelo de MEF foi desenvolvido a partir de uma tomografia computadorizada de uma deformidade hallux valgus (HV) moderada. Cinco configurações diferentes de parafusos foram testadas. A tensão máxima nos metatarsos menores de cada configuração de parafuso, em cargas fisiológicas e suprafisiológicas, foi medida.

**Resultados:**

Os metatarsos menores receberam as menores cargas quando a osteotomia do primeiro metatarso foi fixada com um parafuso intramedular e um bicortical, com carga de tração entre 30 e 70 MPa em cargas fisiológicas e 50 a 350 MPa em cargas suprafisiológicas. Em todas as técnicas de fixação, o 2 ^o^ e o 4 ^o^ metatarsos receberam as maiores cargas, especialmente nos grupos 3 (2 parafusos bicorticais) e 5 (apenas 1 parafuso bicortical), com valores de até 230 e 600 MPa em cargas fisiológicas e suprafisiológicas, respectivamente. Independentemente da técnica de fixação, a região dos metatarsos menores submetida à maior carga foi a diáfise.

**Conclusão:**

Após a cirurgia MICA para correção de HV, houve aumento das forças de tensão nos metatarsos menores, especialmente no segundo e quarto. A técnica de fixação do primeiro metatarso com um parafuso bicortical e um intramedular apresentou os menores valores de carga nos metatarsos menores. Além disso, em cargas fisiológicas e suprafisiológicas, independentemente da técnica, as forças concentraram-se principalmente na diáfise metatársica.

## Abstract (en)

**Objective:**

To analyze the biomechanical consequences on the lesser metatarsals using different screw configurations for fixation of the minimally-invasive Chevron-Akin (MICA) osteotomy, through the finite element method (FEM).

**Methods:**

A FEM model was developed from a computed tomography scan of a moderate HV deformity. Five different screw configurations were tested. We measured the maximal tension in the lesser metatarsals for each screw configurations, in physiological and supraphysiological loads.

**Results:**

The lesser metatarsals received the lowest loads when the first metatarsal osteotomy was fixed with one intramedullary and one bicortical screw, with tensile load values varying between 30 and 70 MPa in physiological loads, and 50 to 350 MPa in supraphysiological loads. In all fixing techniques, the 2nd and 4th metatarsals received the highest loads, especially in groups 3 (2 bicortical screws) and 5 (only 1 bicortical screw), with values reaching up to 230 and 600 MPa in physiological and supraphysiological loads, respectively. Regardless of the fixation technique, the region of the lesser metatarsals that received the most load was the diaphysis.

**Conclusion:**

After MICA surgery to correct HV, there is an increase in tension forces on the lesser metatarsals, especially the second and fourth. The technique of fixing the first metatarsal with one bicortical and one intramedullary screw showed the lowest values on the lesser metatarsals load. Furthermore, for physiological and supraphysiological loads, independently of the technique, the forces were concentrated mainly on the metatarsal shaft.

## Introdução

O termo hallux valgus (HV) refere-se a uma deformidade tridimensional (3D) complexa, composta pelo desvio medial do primeiro metatarso e desvio lateral do hálux. Embora sua etiologia seja multifatorial e ainda não compreendida por completo, essa doença é muito comum na população, especialmente em mulheres. 1 Essas deformidades no primeiro raio podem provocar diversas alterações na biomecânica da marcha e causar sobrecarga mecânica no antepé, dependendo da gravidade do HV. 2 3

O tratamento definitivo do HV é cirúrgico. Entretanto, há diversas técnicas descritas na literatura. Nos últimos tempos, as técnicas minimamente invasivas vêm ganhando popularidade devido ao seu potencial de correção de deformidades, baixa morbidade, recuperação mais rápida e menor custo. 1

Vários estudos destacaram os resultados da técnica de terceira geração, denominada osteotomia minimamente invasiva de Chevron e Akin (MICA, do inglês *minimally-invasive Chevron-Akin* ) para correção do HV. 4 Sabe-se que uma das possíveis complicações após a cirurgia de HV é a metatarsalgia de transferência, causada principalmente pelo encurtamento excessivo e insuficiência do primeiro metatarso. 5 6 Outra preocupação é a escolha do tipo de fixação do primeiro metatarso e sua influência biomecânica no pé. Embora a fixação MICA clássica utilize dois parafusos (um bicortical proximal e um intramedular distal), alguns autores descrevem modificações utilizando apenas um parafuso. 7 8 Entretanto, poucos estudos investigaram a carga no antepé após a correção do HV usando essa técnica. 9 10

O modelo dos elementos finitos (MEF) tem sido utilizado para avaliação da biomecânica do pé e do tornozelo em diversas situações. Por meio de predefinições validadas, 11 é possível simular doenças ou procedimentos cirúrgicos e, assim, avaliar os resultados biomecânicos de forma eficaz. 12 Um estudo anterior 13 com análise por MEF demonstrou que, após a osteotomia em Chevron do primeiro metatarso, o primeiro raio recebeu menos pressão com desvios de 2 a 4 mm e maior pressão com desvios de 6 mm. Enquanto isso, o segundo raio recebeu menos pressão em todos os graus de translação e os demais metatarsos receberam maior pressão, independentemente do grau de translação do primeiro metatarso. Apesar de avaliar diferentes graus de translação da osteotomia, este estudo não analisou o comportamento do primeiro metatarso em diferentes técnicas de fixação. 13

O objetivo deste estudo foi analisar as consequências biomecânicas nos metatarsos menores após cirurgia de MICA para correção de HV, com diferentes técnicas de fixação da osteotomia, por meio do MEF.

## Métodos

### Características Dimensionais e Técnica de Inserção de Parafusos

**Fig. 1:**
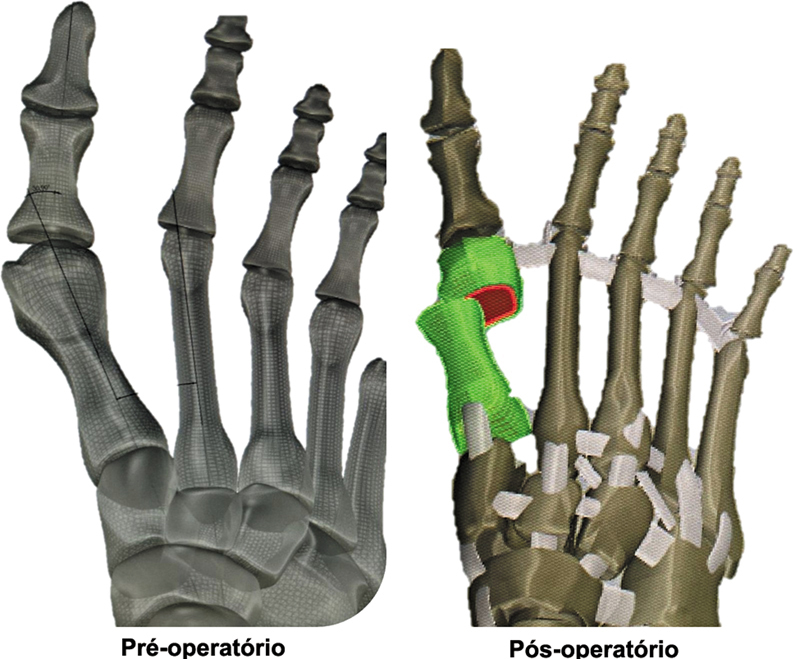
Representação gráfica do modelo simulado pré- e pós-operatório para análise de elementos finitos. Fonte: Lewis et al. 10

Os implantes foram aplicados como indicado pelo fabricante (Novastep), de acordo com suas características dimensionais. A osteotomia MICA foi realizada na base da abertura da metáfise distal/colo do primeiro metatarso, de acordo com a técnica original descrita por Redfern e Vernois. 14 A cabeça do primeiro metatarso foi transladada lateralmente em 75%, com obtenção de ângulo de HV (HVA) de 5° e ângulo intermetatarsal (IMA) de 4° ( Fig. 1 ).

**Fig. 2:**
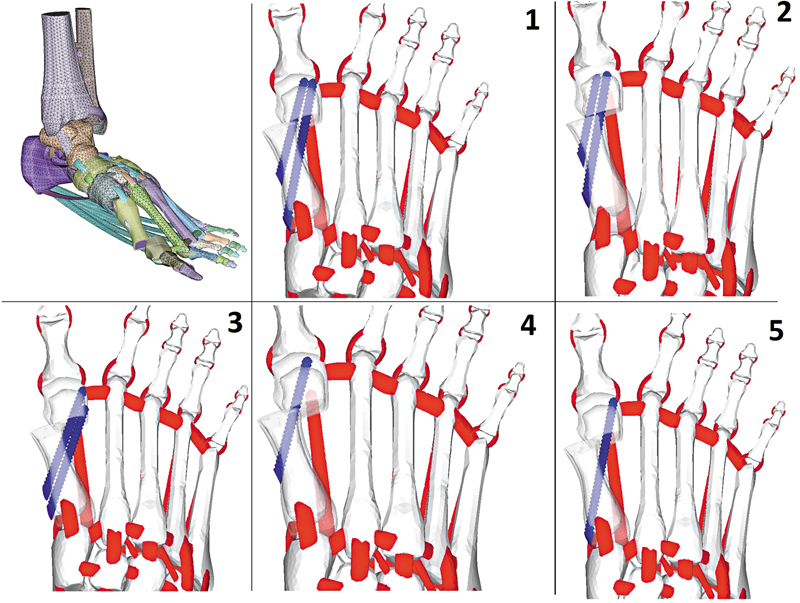
Configuração de fixação do parafuso testada pelo modelo de análise de elementos finitos: grupo 1, um parafuso bicortical e um parafuso intramedular (MICA); grupo 2, dois parafusos intramedulares; grupo 3, dois parafusos bicorticais; grupo 4, um parafuso intramedular; e grupo 5, um parafuso cortical. Fonte: Lewis et al. 10

Cinco grupos foram categorizados com base na técnica utilizada para a fixação da osteotomia MICA. No grupo 1, denominado MICA, a fixação da osteotomia utilizou dois parafusos, um bicortical e um monocortical (intramedular); no grupo 2, dois parafusos intramedulares; no grupo 3, dois parafusos bicorticais; no grupo 4, apenas um parafuso intramedular; e no grupo 5, apenas um parafuso bicortical ( Fig. 2 ).

### Preparação Biocad

O programa Rhinoceros 6 (Robert McNeel & Associates) criou os modelos virtuais tridimensionais de cada sistema (osso e parafusos). A análise de elementos finitos foi realizada com o programa SimLab (HyperWorks), aplicando o solucionador Optistruct (Altair Engineering Inc.). Imagens de tomografia computadorizada (TC) foram obtidas do pé esquerdo de uma mulher de 46 anos, com deformidade moderada do HV (HVA: 30°; e IMA: 14°), sem outras deformidades. As imagens bidimensionais de TC foram usadas para a reconstrução 3D da estrutura anatômica da geometria da superfície do pé pelos programas InVesalius (Centro de Tecnologia da Informação Renato Archer) e STereo Lithography (STL, 3D Systems Inc.). O tomógrafo utilizado foi o Emotion (16 canais, Siemens Healthineers) com intervalo de corte de 2 mm. O estudo seguiu as diretrizes de “Considerations for Reporting Finite Element Analysis Studies in Biomechanics”. 15

### Simulação

O MEF foi usado para simular a carga sobre os metatarsos após a fixação MICA com cinco técnicas diferentes. Primeiro, os arquivos foram importados para o software SimLab e cada parte dos modelos digitais foi identificada, garantindo a manutenção do tamanho do elemento para evitar quaisquer problemas de contato entre as diferentes partes durante as simulações.

A discretização do domínio geométrico foi realizada usando elementos tetraédricos de segunda ordem com comprimento médio de borda de 3 mm nos ossos corticais e trabeculares, 0,5 mm na área, 2 mm nos ligamentos e refinamento nas regiões de contato com tamanho médio de borda de 0,8 mm. Todos os tecidos foram definidos como homogêneos, isotrópicos e linearmente elásticos. Um elemento tetraédrico foi adotado para formar as malhas. As propriedades dos materiais usados para as simulações foram o módulo de Young e o coeficiente de Poisson, seguindo um estudo anterior. 1 Uma análise padrão de sensibilidade da malha foi realizada para assegurar que a densidade usada no MEF fosse suficiente para atingir resultados numéricos convergentes, sem necessidade de maior refinamento.

### Condições de Contorno e Carga

Considerando as condições fisiológicas, com o antepé e o retropé fixos, uma força de reação do solo (FRS) vertical foi aplicada ao mediopé. Não houve aplicação de carga nos eixos X e Y. A força vertical ascendente do tendão calcâneo também foi criada com metade do valor da FRS. Todos os modelos foram testados em duas condições fisiológicas diferentes (150 e 300 N). O MEF foi aplicado para medir a tensão máxima em cada um dos metatarsos menores.

## Resultados

**Fig. 3:**
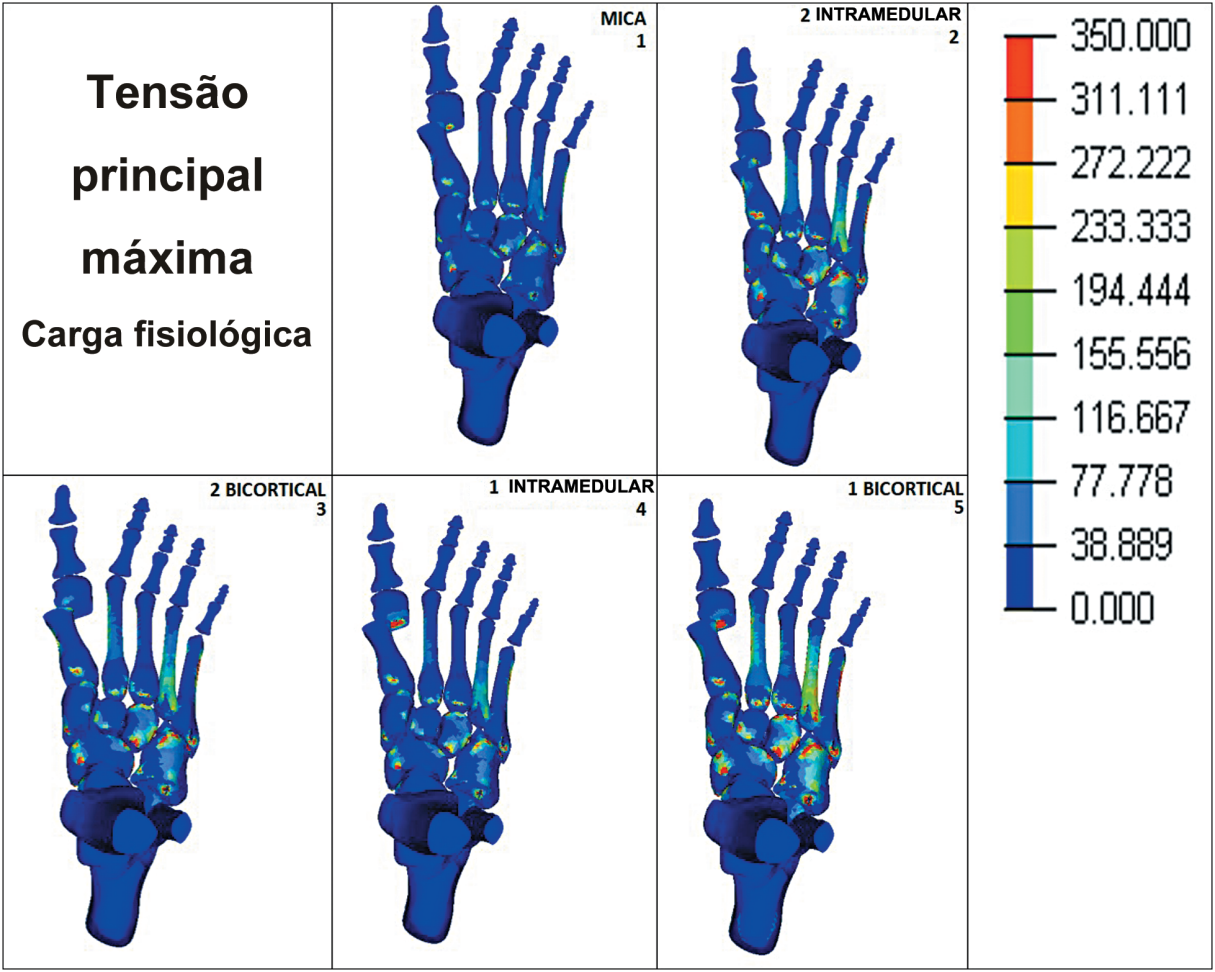
Tensão principal máxima em carga fisiológica (150 N) em 5 diferentes técnicas de fixação.

Observou-se que, quando submetidos à carga fisiológica, os diferentes métodos de fixação da osteotomia do primeiro metatarso apresentaram tensões principais máximas (forças de tração) diferentes nos metatarsos menores. O grupo 1 apresentou os menores valores, entre cerca de 30 e 70 MPa. Os maiores valores foram observados no segundo e no quarto metatarsos dos grupos 2, 3 e, principalmente, do grupo 5, cujos valores foram de aproximadamente 150 MPa no segundo metatarso e 230 MPa no quarto. No grupo 4, o segundo e o quarto metatarsos apresentaram tensões máximas de cerca de 70 e 115 MPa, respectivamente ( Fig. 3 ).

**Fig. 4:**
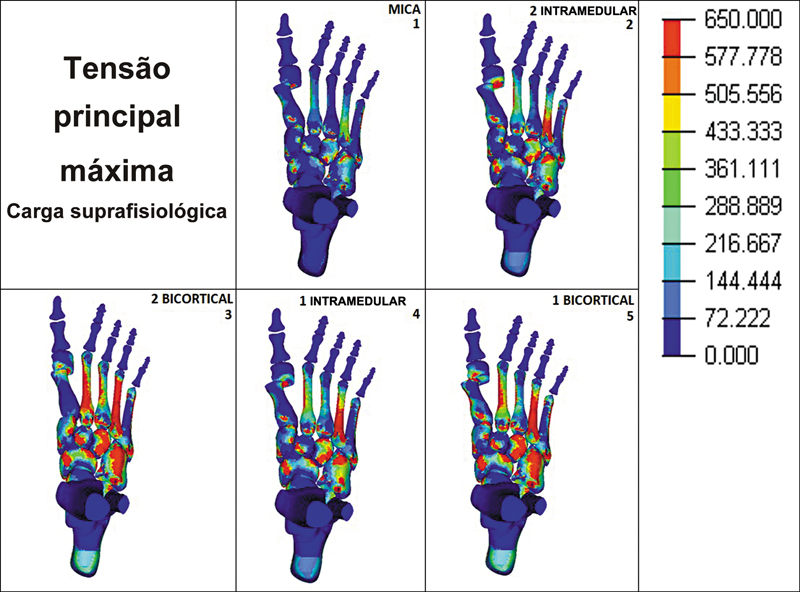
Tensão principal máxima em carga suprafisiológica (300 N) em 5 diferentes técnicas de fixação.

Quando os MEFs foram submetidos à carga suprafisiológica, observou-se que a tensão máxima foi maior nos metatarsos menores, principalmente no segundo e no quarto metatarsos. Os grupos 3 e 5 apresentaram valores de cerca de 600 MPa no segundo e no quarto metatarsos. Nos grupos 2 e 4, o segundo e o quarto metatarsos apresentaram tensão máxima de cerca de 300 e 600 MPa, respectivamente. No grupo 1 (MICA), os metatarsos menores apresentaram os menores valores de tensão máxima, variando entre cerca de 50 e 350 MPa ( Fig. 4 ).

Por fim, observou-se que, tanto com cargas fisiológicas quanto suprafisiológicas, a diáfise foi a região dos metatarsos menores que recebeu a maior concentração de cargas, independentemente da técnica utilizada para fixação da osteotomia do primeiro metatarso.

## Discussão

Neste estudo, avaliamos, por meio do MEF, a carga sobre os metatarsos após a cirurgia MICA para correção de HV com diferentes técnicas de fixação da osteotomia. Os principais achados foram os menores valores apresentados nos metatarsos menores do grupo 1 sob cargas fisiológicas e suprafisiológicas. Além disso, com ambas as cargas, todas as outras técnicas de fixação apresentaram os maiores valores no segundo e no quarto metatarsos, em especial nos grupos 3 e 5. Esses resultados confirmam a relevância do uso de um parafuso intramedular, em conjunto com um bicortical, o que pode evitar a sobrecarga sobre o metatarso menor.

A osteotomia MICA é uma técnica utilizada para correção da HV que tem resultados promissores, com bons desfechos radiológicos e clínicos. 4 Um estudo prospectivo 9 com 31 pés de 25 pacientes com HV moderada e grave sem metatarsalgia, usou pedografia para avaliar as transferências de carga para os metatarsos menores após a correção cirúrgica de HV, com a técnica minimamente invasiva de quarta geração. O estudo demonstrou uma redução nas cargas sobre o primeiro raio, com uma diminuição na carga sobre o metatarso central, 3 meses após a cirurgia. Os autores concluíram que a técnica pode não prevenir ou corrigir a metatarsalgia. Por outro lado, em nosso estudo, a técnica MICA para correção de HV apresentou altas cargas nos metatarsos menores, especialmente no segundo e quarto.

Estudos anteriores investigaram a fixação do primeiro metatarso após osteotomia MICA com apenas um parafuso bicortical, demonstrando bons resultados clínicos e radiológicos. 7 8 No entanto, nenhum desses estudos analisou a transferência de carga para os metatarsos menores no período pós-operatório. Em nosso estudo, o uso de um ou dois parafusos intramedulares (grupos 2 e 4) ou bicorticais (grupos 3 e 5) gerou resultados semelhantes, com cargas maiores no segundo e quarto metatarsos. Tais transferências de carga para os metatarsos menores não foram observadas na técnica MICA, que apresentou os melhores resultados biomecânicos. Isso sugere que a adição de um segundo parafuso na mesma posição (ou seja, intramedular ou bicortical) não é vantajosa em relação à transferência de carga para os metatarsos menores. Portanto, o uso de dois parafusos com posicionamentos diferentes é ideal, conforme a técnica MICA.

Diversas complicações foram descritas após a osteotomia do primeiro metatarso para correção da deformidade em HV. Uma delas é a metatarsalgia de transferência, com ocorrência estimada de 5,4% em cirurgias percutâneas. 16 Uma de suas prováveis causas seria a insuficiência do primeiro raio, seja por encurtamento excessivo ou fixação em posição de dorsiflexão. 5 6 17 Em nosso estudo, após a construção da osteotomia por MEF, não houve desvio no plano sagital ou encurtamento. Esse fato poderia explicar o aumento da tensão nos metatarsos menores. Além disso, curiosamente, em todos os tipos de fixação, as cargas se concentraram mais na diáfise do metatarso menor, o que pode ocasionar metatarsalgia de transferência, fratura por estresse ou deformidades nos dedos (dedos em garra). Portanto, os cirurgiões devem estar atentos ao encurtamento excessivo e aos desvios da cabeça do primeiro metatarso no plano sagital, evitando possíveis sobrecargas sobre os metatarsos.

Nosso estudo tem diversas limitações, em sua maioria relacionadas à análise por MEF. Primeiro, nem todos os leitores estão familiarizados com esta ferramenta de análise. Também consideramos a anatomia de um único pé, com translação de apenas 75% da cabeça do primeiro metatarso e um tipo de modelo de carga. Além disso, para fins de modelagem, considerou-se que as propriedades mecânicas dos materiais, incluindo osso cortical, osso trabecular, ligamentos e sínteses, eram elásticas lineares contínuas, isotrópicas e uniformes.

A precisão dos resultados do MEF depende dos parâmetros inseridos e das suposições feitas durante o desenvolvimento do modelo. Logo, propriedades imprecisas do material podem levar a resultados divergentes. Simplificações na modelagem, como assumir o comportamento linear do material ou a anatomia limitada, podem afetar a precisão das previsões. Também é importante destacar que uma simulação dinâmica pode originar resultados diferentes, diferentemente da simulação estática realizada neste estudo. É impossível considerar a variabilidade interindividual e outros mecanismos compensatórios in vivo. Este modelo também não considerou diferentes configurações de osteotomia ou desvios percentuais da cabeça do metatarso. Portanto, os resultados aqui apresentados podem diferir daqueles obtidos em estudos in vivo. Porém, como nosso objetivo era avaliar apenas os métodos de fixação, tentamos recriar um teste removendo as variações encontradas em estudos em humanos ou cadáveres.

## Conclusão

No presente estudo, a análise por MEF mostrou que, após a osteotomia MICA para correção de HV, há um aumento nas forças de tensão nos metatarsos menores, especialmente no segundo e quarto. A técnica de fixação do primeiro metatarso com um parafuso bicortical e um intramedular apresentou os menores valores de cargas nos metatarsos menores. Além disso, sob cargas fisiológicas e suprafisiológicas, independentemente da técnica, as forças concentraram-se mais na diáfise do metatarso.
